# A Fault Detection Method for Electrohydraulic Switch Machine Based on Oil-Pressure-Signal-Sectionalized Feature Extraction

**DOI:** 10.3390/e24070848

**Published:** 2022-06-21

**Authors:** Qingzhou Meng, Weigang Wen, Yihao Bai, Yang Liu

**Affiliations:** School of Mechanical, Electronic and Control Engineering, Beijing Jiaotong University, Beijing 100044, China; 20121246@bjtu.edu.cn (Q.M.); 19116001@bjtu.edu.cn (Y.B.); yliu16@bjtu.edu.cn (Y.L.)

**Keywords:** electrohydraulic switch machine, oil pressure signal, sectionalized feature extraction, fault detection, mRMR, unsupervised clustering

## Abstract

A turnout switch machine is key equipment in a railway, and its fault condition has an enormous impact on the safety of train operation. Electrohydraulic switch machines are increasingly used in high-speed railways, and how to extract effective fault features from their working condition monitoring signal is a difficult problem. This paper focuses on the sectionalized feature extraction method of the oil pressure signal of the electrohydraulic switch machine and realizes the fault detection of the switch machine based on this method. First, the oil pressure signal is divided into three stages according to the working principle and action process of the switch machine, and multiple features of each stage are extracted. Then the max-relevance and min-redundancy (mRMR) algorithm is applied to select the effective features. Finally, the mini batch k-means method is used to achieve unsupervised fault diagnosis. Through experimental verification, this method can not only derive the best sectionalization mode and feature types of the oil pressure signal, but also achieve the fault diagnosis and the prediction of the status of the electrohydraulic switch machine.

## 1. Introduction

In a railway, a switch machine is track switch equipment that makes train transfer from one track to another, and it is an important signal device to ensure the train operation safety. A switch machine is often eroded under a poor working environment with high intensity and heavy load, which leads to fault occurrence. The fault of switch machines may cause serious accidents, such as train derailment and capsizing, resulting in heavy casualties and property losses. Fault detection of switch machines is very important for railway transportation safety. However, for a long time, the fault detection of switch machines mainly depends on the expert knowledge and experience of railway workers, or adopts a simple threshold setting method, which not only are inefficient but also bring a heavy workload to railway workers, which often leads to misjudgment.

Recently, many fault diagnosis methods combining a support vector machine (SVM), fuzzy logic system, artificial neural network (ANN), and others have been constantly appearing. Asada [1] proposed an effective approach for accurately classifying several fault modes combined with a wavelet transform and support vector machines to detect faults of an AC point machine. In [2], support vector machines with a Gaussian kernel were used to diagnose a fault of an electric switch machine, which verified the features obtained with principal components analysis (PCA) getting better results than the existing features. Moreover, an SVM-based fault detection approach was proposed to identify the fault states of a switch machine based on the electric current curve, and the envelope and morpheme match algorithm were applied to predict the fault of a switch machine in this approach [3]. The type-1 and singleton fuzzy logic system trained by the conjugate gradient method was proposed by de Aguiar [4] to realize fault diagnosis by different classifiers based on the current signal of an electric switch machine, which could offer a higher convergence rate and performance. Further, de Aguiar [5] used the set-membership concept derived from the adaptive filter theory in the type-1 and singleton/nonsingleton fuzzy logic systems so that the model convergence speed was improved and computation complexity was reduced, and then he demonstrated that the upper and lower singleton type-2 fuzzy logic system was a more effective classifier for an electric switch machine fault [6]. The long- and short-term memory (LSTM) and the deep wavelet scattering transform (DWST) were explored for classifying various switch degradations, and the feasibility of a dataset captured under the service conditions was demonstrated in [7]. A hybrid fault diagnosis (HFD) method was adopted to identify a fault based on the current curves of a railway switch machine in [8]. A locally connected autoencoder was employed to automatically capture high-order features in order to solve the fault diagnosis problem with no training steps based on the current signal of an electric point machine [9]. DAG-SVMs were applied to intelligently detect a fault after extracting characteristics based on the action current signal of an electric switch machine, and the experiment showed that the accuracy of classification after Kalman filter pretreatment was better than that of direct classification in [10]. The artificial intelligent methods, such as RNN (recurrent neural network), CNN (convolutional neural network), autoencoder, and other deep learning methods, were adopted for feature extraction and fault diagnosis in industrial fields [11,12,13]. More and more intelligent fault diagnosis methods were applied in various engineering areas, which could achieve diagnosis accurately. Additionally, the methods provided a reliable basis for fault detection of the switch machine.

The intelligent fault diagnosis methods were also applied to some special electric switch machines, such as ZD6 and S700K. The similarity function was defined by the Fréchet distance, action current template curves were built, and the fault diagnosis method according to the greatest curve similarity was constructed for a ZD6 switch machine in [14]. In [15], the railway turnout intelligent fault detection algorithm was proposed based on a BP neural network by analyzing the characteristics of action current curves for a ZD6 switch machine. A senior Bayesian model based on rough set theory was applied to detect the fault of an S700K switch machine, which effectively enhanced the speed and accuracy of fault diagnosis [16]. The wavelet packet energy entropy was used for switch machine fault detection based on the three-phase alternating current of an S700K switch machine [17]. Grey correlation analysis and a neural network were combined to obtain better detection results based on power curves of an S700K electric switch machine [18]. A fuzzy clustering analysis method based on the action power curve was proposed to achieve the fault detection of an S700K switch machine, which could accurately extract fault features and support simultaneous detection of multiple faults [19]. In [20], ensemble empirical mode decomposition (EEMD) based on the power curve of an S700K switch machine was proposed to decompose a signal, and the fuzzy clustering analysis algorithm was used to realize fault classification, which were more fully characterized fault signals. Variational mode decomposition (VMD) and the kernel fuzzy c-means (KFCM) clustering algorithm were employed to classify the fault types of an S700K switch machine based on the power curve, whose better classification results were obtained by adding kernel functions [21]. FCM and hidden semi-Markov models (HSMMs) were combined to quickly and accurately identify the health status based on the power data of an S700K switch machine [22].

However, there are other types of switch machine, which are applied in different railway passenger stations, freight stations, and marshalling stations. An electrohydraulic switch machine is a new type of switch machine that has appeared in China since the 1980s, which is suitable for the development trend of a high-speed railway. An electrohydraulic switch machine uses hydraulic transmission, and hydraulic oil is generally utilized as a working medium. Additionally, the oil pressure signal contains a lot of useful information about operation and the fault status of an electrohydraulic switch machine. Zhou [23] applied grey correlation theory to the intelligent fault detection of turnouts based on the oil pressure signal of a ZYJ7 electrohydraulic switch machine. For the existing fault detection, the expert system [24] is hard for acquiring knowledge and needs a lot of prior knowledge of railway staff; the Kalman filtering method [25] can only be successful in a part of the dataset; a reliable and reasonable prior probability has to be provided for a Bayesian network [26], in which determination is very difficult; a support vector machine [3] is a binary classifier in principle, which is very sensitive to feature selection; and a neural network [7] needs numerous samples for training to avoid misdetection. However, unsupervised clustering methods can support multiple fault detections and effectively improve performance, which do not need to be trained and be provided with many prior parameters. Most of the literature has focused on the fault detection of electric switch machines based on the current or power signal. However, there is a pressing need for feature extraction and fault detection research works for electrohydraulic switch machines with more application and promotion of speed-up turnouts in the future. The features from oil pressure signal time series can effectively provide information reflecting the fault status and obtain a better detection effect for an electrohydraulic switch machine. However, the oil pressure signal of an electrohydraulic switch machine includes plenty of fault characteristics, which is nonstationary and difficult to extract. Besides, existing methods of extracting a feature directly from the whole signal and sectionalizing averagely a feature extraction are not able to extract effective feature information from the signal.

For the above reasons, a novel fault detection method for an electrohydraulic switch machine based on the sectionalized feature extraction according to the best time ratio from the oil pressure signal is proposed in this paper. The rest of the paper is organized as follows: Section 2 describes the principles and the framework of the proposed approach. The effectiveness and accuracy of the fault detection method based on sectionalized feature extraction are illustrated by experiments in Section 3. Finally, some conclusions are summarized in Section 4.

## 2. Materials and Methods

### 2.1. Sectionalized Feature Extraction

**Figure 1:**
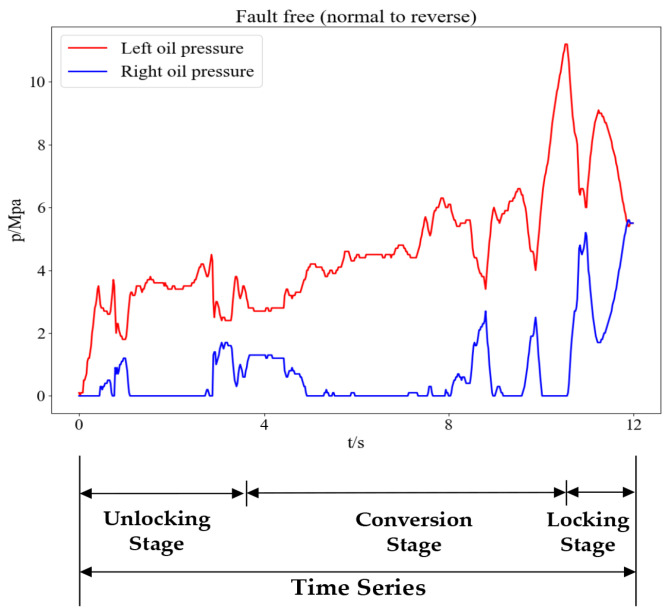
Three stages of the oil pressure signal.

In order to extract the features of the different action states in the switching process, the original oil pressure time series is divided into multiple intervals, and the feature parameters of each interval are calculated. In contrast to the method of directly extracting the statistical parameters of the entire oil pressure curve, this method of sectionalized feature extraction allows for the preservation of both the detailed feature information for each interval and the entire signal. According to the working principle of the electrohydraulic switch machine, the whole switching process is mainly divided into three stages: unlocking, conversion, and locking. Each stage is completed by a different working principle, so it makes sense to study the time ratio of the oil pressure signal sectionalization. Based on the working principle and practical experience, this paper proposes the “three-stage method” for the sectionalization of the oil pressure signal, with a sectionalized time ratio of 30%–60%–10%, which divides the entire oil pressure signal into three stages according to this time ratio, as shown in Figure 1.

The process of feature extraction in each interval is to map the original oil pressure signals into the fault feature space. The time domain features can reflect the energy distribution and various effects of the signal and effectively represent the action states of the switch machine. In order to describe the state of the oil pressure signal for each interval, the following statistical features are used in this paper, and their calculation formulas are shown in Table 1.

We suppose that the original oil pressure signal is $x\left(i\right)$, where $i=1,2,\ldots ,N_{s},{\;N}_{s}$ denotes the signal length. Δt means the sampling interval. The mean value $\bar{x}$ represents the average magnitude of the signal segment. The variance (Var) describes the fluctuation range of the signal near its mean value. The kurtosis (Ku) can well describe the distribution pattern of signal variables. The peak-to-average ratio (PAR) reflects the extreme degree of the peak in each signal interval. The impulse indicator (IM) is used to express the accumulation effect of the oil pressure signal. These feature indicators describe the characteristics of the original oil pressure signal from different aspects. They can quantitatively reflect the degree of signal dispersion, changes, and asymmetry in each interval and play an important role in the fault detection of an electrohydraulic switch machine.

**Table 1:** Calculation formulas for extracting five types of time series features.

Feature Type	Calculation Formula	Significance of Feature
Mean	$\bar{x}=\frac{1}{N_{s}}\sum_{i=1}^{N_{s}}x\left(i\right)$	Reflect the mean amplitude
Variance (Var)	$\sigma^{2}=\frac{1}{N_{s}-1}\sum_{i=1}^{N_{s}}{\left(x(i\right)-\bar{x})}^{2}$	Represent the deviant trend of signal near the mean value
Kurtosis (Ku)	$K_{u}=\frac{\sum_{i=1}^{N_{s}}{\left(x(i\right)-\bar{x})}^{4}}{{(N}_{s}-{1)\sigma }^{3}}$	The distribution pattern of signal variables
Peak-to-average ratio (PAR)	$\mathrm{PAR}=\frac{\sqrt{N_{s}}\;\max|x(i)|}{\sum_{i=1}^{N_{s}}|x\left(i\right)|}$	The extreme degree of peak value in the signal segment
Impulse (IM)	$\mathrm{IM}=\sum_{i=1}^{N_{s}}x(i)\Delta t$	The cumulative effect of signal

### 2.2. Feature Smoothing

The oil pressure feature set inevitably contains a lot of noise and the random fluctuation caused by the non-stationarity of the oil pump, internal equipment, and environment. In addition, some of the extracted feature values change drastically, while others vary with small numerical fluctuations, which leads to the features with large fluctuations playing a more critical role than the others. Locally weighted regression (LWR) smoothing is a regression method used to smoothen a volatile time series. It is a nonparametric method where least squares regression is performed in localized subsets, which makes it a suitable candidate for smoothing any fluctuating numerical vector. LWR is superior to ordinary smoothing methods in filtering random changes and revealing feature trends [27].

The process of LWR smoothing is simply defined as follows: First, the feature data are divided into small intervals with a sliding window, linear fitting is performed through weighted least squares within the interval, and the fitted value of the focal point in each interval is obtained. Then, this process is repeated continuously with the window sliding for all the feature data. Finally, the centers of these regression curves are connected to form a complete regression curve. LWR can remove error points polluted by noise and random fluctuation. LWR adds weight to the ordinary least squares, and the points near the fitting point should have a greater impact on the fitting value. Let us assume that we use LWR to smoothly fit a curve of ($y_{j},z_{j}$) in each window, where j = 1, …, n. The weighted linear regression is calculated according to the loss function L of Equation (1):

(1) $$L(\theta ,h)=\overset{n}{\underset{j=1}{\sum }}w_{j}{\left(z_{j}-{\theta y}_{j}-h\right)}^{2}$$

where $\theta =\frac{\sum w_{j}^{2}(y-\bar{y})(z-\bar{z})}{\sum w_{j}^{2}{(y-\bar{y})}^{2}}$ denotes the gradient, and $h=\bar{z}-\theta \bar{y}$ denotes the constant for each linear fitting.

It is necessary to determine the distances from the points within each interval to the fitting point $\left|y_{0}-y_{j}\right|$ and find the largest distance ${\max(y}_{j})$. The weight can be obtained according to Equation (2):

(2) $$w_{j}(y_{0})=W\left(\frac{\left|y_{0}-y_{j}\right|}{\max(y_{j})}\right)$$

The function W can choose a quadratic function (B function) or cubic function (C function). The C function is commonly used in the first iteration, and the B function in the second iteration [28]. The calculation formula of the C function is formulated as follows:

(3) $$W\left(\rho \right)=\left\{\begin{array}{cc} \;{\left(1-\rho^{3}\right)}^{3}, & \mathrm{for}\left|\rho \right|<1\\ \;0, & \mathrm{for}\left|\rho \right|\geq 1 \end{array}\right.$$

where ρ is described as:

(4) $$\rho =\frac{\left|y-y_{j}\right|}{\max\left(y_{j}\right)}$$

LWR is employed to remove drastic changes, noise, and spike information and to improve the accuracy of fault classification.

### 2.3. Min-Max Normalization Processing

There are different unit dimension features that cannot make an evaluation in such a multidimension system. Therefore, in order to ensure the reliability of the results, it is necessary to normalize the oil pressure feature set. The normalization processing scales the data to a specific interval within a certain range. The purpose is to remove the unit limit of data and transform all the features into a nondimensional data. Here, we use min–max normalization to map the smoothed oil pressure feature set to domain (0–1). Min–max normalization can be calculated as:

(5) $$T=\frac{Q-\min\left(Q\right)}{\max\left(Q\right)-\min\left(Q\right)}$$

where Q means the smoothed oil pressure feature set. T denotes the min–max normalization result of Q.

### 2.4. Feature Reduction Based on the Max-Relevance and Min-Redundancy Algorithm

To reduce the computational complexity, traditional methods are to directly reduce the dimensions of the feature. Related methods include local linear embedding (LLE), local preserving projection (LPP), principal component analysis (PCA), and so on. These methods reduce the feature’s dimensions through space mapping [29]. In [30,31], the authors defined feature relevance and divided features into three categories: strong relevance features, weak relevance features, and irrelevant features. Among the above features, strong relevance features must be retained; otherwise, they will seriously affect the classification performance. The weak relevance features are not required, but sometimes they are necessary, so they need to be selected according to the situation [32]. Nevertheless, the irrelevant features need not be kept, instead, they need to be removed.

If two features are completely related, they are mutually redundant features. We brought the idea of the max-relevance and min-redundancy (mRMR) algorithm in [33]; that is, the feature with the highest relevance with the target classification label c is selected, and then features with high redundancy are removed. Here, the relevance usually uses mutual information (MI) as a common indicator to define the relevance of variables. Relevance is the average value of the mutual information (MI) between the feature $A_{u}$ and the categorical label c. $I\left(A_{u};c\right)$ is used to represent the mutual information. The max-relevance can be obtained by the following equation:

(6) $$\max\;D(T),\;D=\frac{1}{\left|T\right|}\underset{A_{u}\in T}{\sum }I\left(A_{u};c\right)$$

where max D refers to the feature set with maximum relevance to the classification label; u is 1,2,…,N; and N represents the number of features after smoothing and normalization.

The features selected by maximum relevance are likely to have some redundant features, the removal of which will have no effect on the classification result but will significantly reduce the computational effort of the classification algorithm. The selection and removal of mutually exclusive features using minimum redundancy is expressed as:

(7) $$\min\;R\left(T\right),\;R=\frac{1}{{\left|T\right|}^{2}}\underset{A_{u},A_{v}\in T}{\sum }I\left(A_{u},A_{v}\right)$$

where min R refers to the feature set with minimum redundancy. $A_{v}$ denotes the features in the feature set, v=1,2,…,N, v≠u. Here, a different algorithm from the traditional mRMR is considered to obtain the best features, integrating the optimization of max D and min R as follows:

(8) $$●\;\max\tau \left(D,R\right),\;\tau \left(D,R\right)=\frac{D}{R+0.01}$$

where $\max\;\tau \left(D,R\right)$ denotes the result of the mRMR algorithm. Adding 0.01 to the R value is to avoid the inability to calculate when R is 0.

### 2.5. Proposed Framework

**Figure 2:**
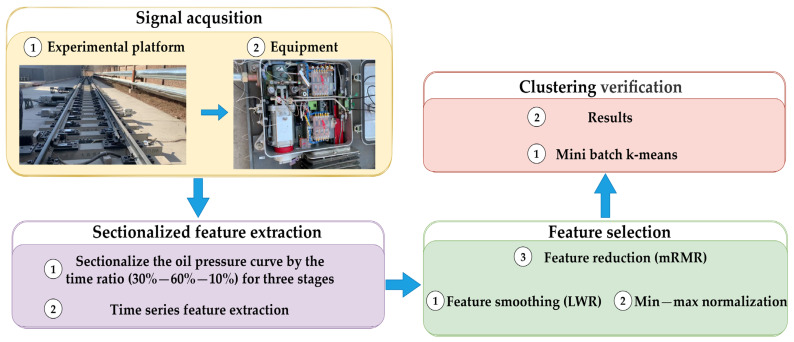
Flow chart of the proposed fault diagnosis method.

The process of fault detection of an electrohydraulic switch machine based on the oil-pressure-signal-sectionalized feature extraction method is illustrated in Figure 2. The process is described as follows:

Step 1: Collecting the oil pressure signals from the left and right sides of the oil cylinder of an electrohydraulic switch machine;
Step 2: Sectionalizing the oil pressure signal according to the time ratio (30%–60%–10%) for three stages;
Step 3: Calculating five types of time domain features of each segment for the oil pressure signal;
Step 4: Smoothing the features by locally weighted regression (LWR) and normalizing the smoothed features by min–max normalization;
Step 5: The max-relevance and min-redundancy (mRMR) algorithm is employed to eliminate redundant features and obtain the optimal fault feature set;
Step 6: The mini batch k-means clustering method is used to achieve the fault detection of the electrohydraulic switch machine.

## 3. Experiments

The faults of the electrohydraulic switch machine being studied in this paper are mainly mechanical faults. Common mechanical faults include mechanical resistance, oil cylinder action not in place, oil cylinder in place with contact point not closed or rebound broken indication, and so on. Several common fault types and cause analysis are shown in Table 2. Meanwhile, the position of a switch machine is classified to normal position and reverse position, that is, from normal position to reserve position and reserve position to normal position. Turnout not locking and not unlocking are two common faults of railway turnouts, because the existence of a stuck phenomenon and the increase in resistance result in the failure of switch machines to work normally.

### 3.1. Data Description

**Figure 3:**
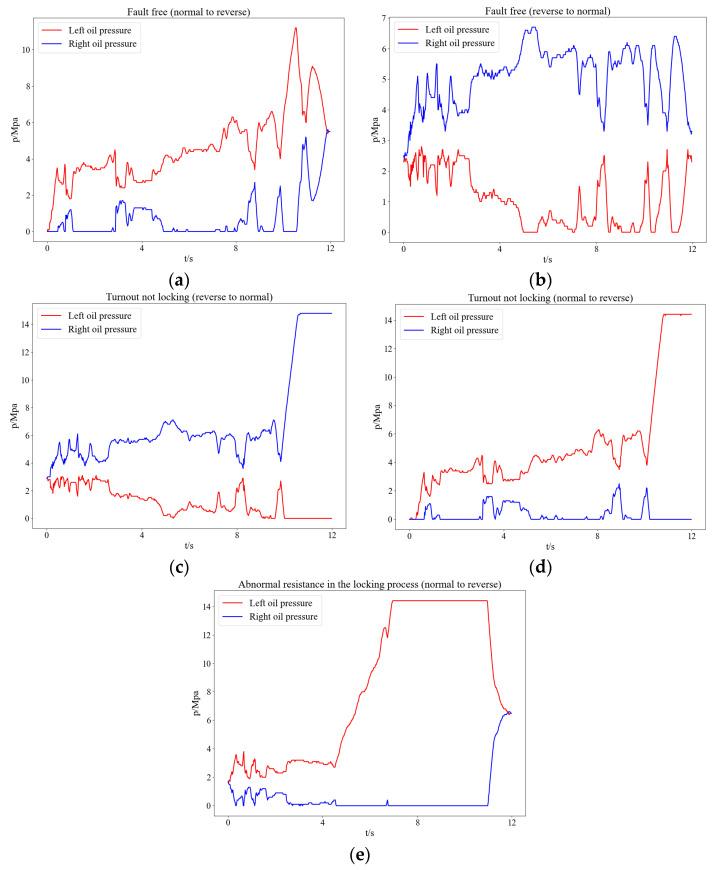
The time domain waveforms of oil pressure signal: (**a**) fault-free (normal to reverse), (**b**) fault-free (reverse to normal), (**c**) turnout not unlocking (reverse to normal), (**d**) turnout not unlocking (normal to reverse), (**e**) abnormal resistance in the locking stage (normal to reverse).

A measuring device was used to collect oil pressure signals from an electrohydraulic switch machine. The measuring device consisted of an acquisition instrument, oil pressure sensors, and a computer. The signals were collected by deploying an oil pressure sensor on the left and right sides of the oil cylinder of the switch machine. The collected data of the sensors were transmitted to the acquisition instrument, which then was sent to the computer. The sensors collected approximately 1000 sets of oil pressure signals during the action process of the electrohydraulic switch machine with a sampling frequency of 50 Hz, an acquisition time of 12 s, and a measurement range of 0 to 14.8 MPa. In the experiments, five types of faults are made: fault-free (normal–reverse), fault-free (reverse–normal), turnout not locking (reverse–normal), turnout not locking (normal–reverse), abnormal resistance in the locking stages (normal–reverse), as shown in Figure 3. The fault labels and corresponding action states of the electrohydraulic switch machine are shown in Table 3.

**Table 3:** Fault labels and action states of an electrohydraulic switch machine.

Fault Labels	Action States
0	Fault-free (normal–reverse)
1	Fault-free (reverse–normal)
2	Turnout not locking (reverse–normal)
3	Turnout not locking (normal–reverse)
4	Abnormal resistance in the locking stage (normal–reverse)

### 3.2. Experiment I

The purpose of this fault clustering experiment was to evaluate the fault identification results of different sectionalized modes of feature extraction. The scheme not only extracted the most important feature types, but also identified the classes of fault clustering. At first, in order to be able to extract more valid information, the sectionalized mode of three stages with the time ratio (30%–60%–10%) and the five feature parameters proposed in Section 2.1 were applied for feature extraction, and in total, 15 × 2 = 30 features were extracted from oil pressure signals of the left and right cylinder, which was named as Dataset I. In order to verify the validity of our method, the following features of different sectionalized types were extracted for comparative analysis. The five features extracted directly from the whole sequence of the oil pressure signal formed Dataset II, and the same features were computed on the two and three oil pressure signal segments that were sectionalized averagely composing Datasets III and IV. Moreover, the features were smoothed and normalized with a uniform smoothing window width of 10, and the max-relevance and min-redundancy (mRMR) algorithm was used to eliminate redundant features and derive the optimal feature set. The extracted feature sets from the four datasets after mRMR are shown in Table 4, Table 5, Table 6 and Table 7 as follows:

Mini batch k-means clustering was used for clustering analysis in the experiments. The traditional k-means algorithm is used to calculate the distance from all sample points to all centers of mass. If the feature dimension of the sample is too large, the k-means algorithm will be very time-consuming. The mini batch k-means algorithm is an optimized variant of the k-means algorithm in which a small subset of data is randomly selected for each training to reduce computation time while optimizing the aim function.

**Figure 4:**
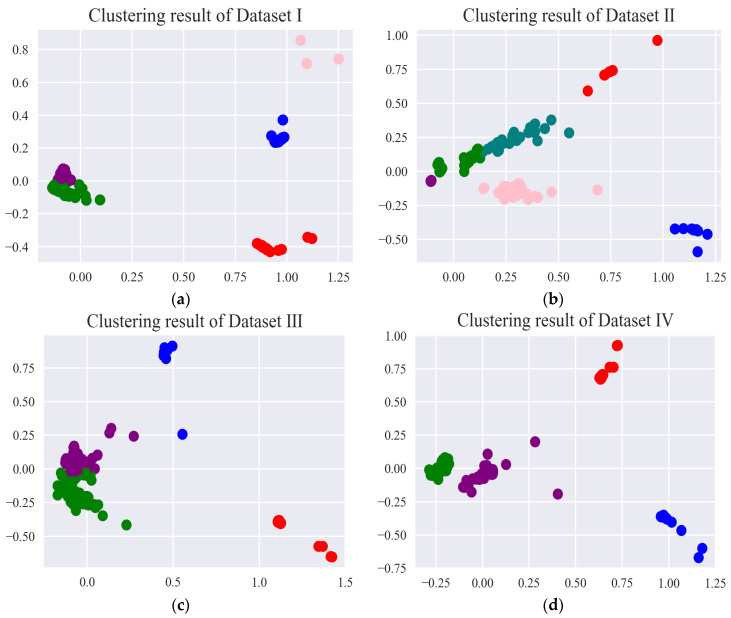
Clustering results of the optimal clustering number for different datasets after mini batch k-means: (**a**) Dataset I, (**b**) Dataset II, (**c**) Dataset III, and (**d**) Dataset IV.

In order to better display data characteristics, PCA was used to reduce the dimension of feature sets to 2. The batch size was set to 100, and the other parameters were fixed in the experiments. Table 8 represents the variations of the DBIs (Davies–Bouldin index) [34] with the number of clusters ranging from 2 to 10 by mini batch k-means for different datasets. The corresponding optimal cluster numbers for each DBI are indicated in bold font. As it is seen in Table 8, only Dataset I determined the right cluster number, and the other datasets were unable to determine the number of the right clusters. After determining the right cluster numbers, the clustering results for different datasets are shown in Figure 4. As it is clear in Figure 4 and Table 8, the proposed method has recognized the five different clusters in Dataset I. It was made clear that this proposed method of sectionalized feature extraction performed particularly well in the visualization of clusters.

The green cluster (fault-free (reverse to normal)) and the purple cluster (fault-free (normal to reverse)) were always close to each other in Figure 4, suggesting that the two clusters represented similar real meanings for the normal state of an electrohydraulic switch machine.

**Figure 5:**
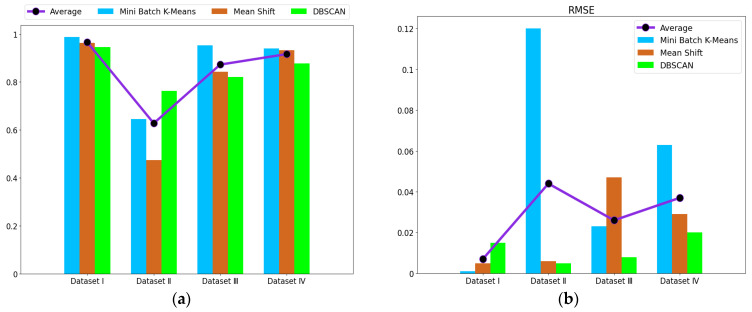
Display of the diagnosis results for different datasets after clustering: (**a**) average of accuracy; (**b**) RMSE.

**Figure 6:**
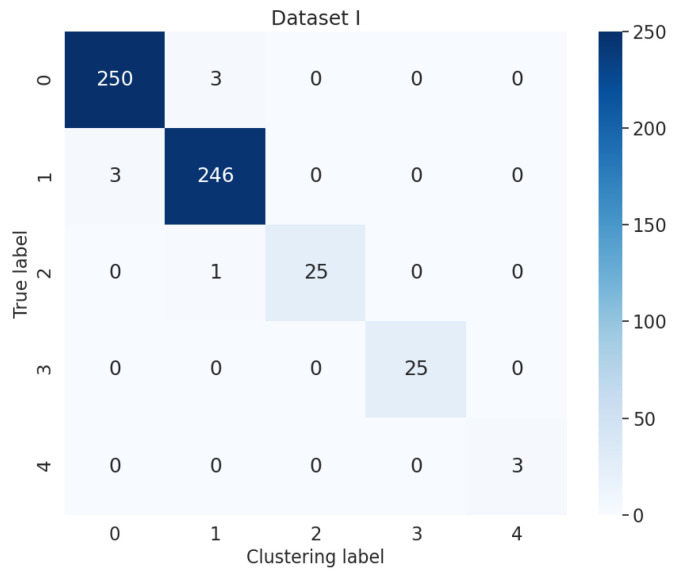
Confusion matrix for Dataset I with the proposed method after mini batch k-means.

Additionally, the external metrics for the accuracy of the results were evaluated. To improve the experiment analysis, we reran mini batch k-means with 10 different initial random seeds, mean shift with 10 different eps, and density-based spatial clustering of applications with noise (DBSCAN) with 10 different bandwidths. Mean shift and DBSCAN were applied to compare the validity of different clustering methods. The average of accuracy and root-mean-square error (RMSE) for different datasets after clustering are shown in Table 9 and Figure 5. The confusion matrix for Dataset I is shown in Figure 6. The proposed method based on mini batch k-means had the best average of accuracy and the lowest RMSE for diagnosis results. This verifies that the sectionalized feature extraction method corresponding to the three stages (30%–60%–10%) has the best clustering performance for fault detection compared with other modes.

**Table 9:** Average of accuracy and RMSE for different datasets after clustering.

Different Datasets	Mini Batch K-Means		Mean Shift		DBSCAN		Average	
	Average of Accuracy (%)	RMSE	Average of Accuracy (%)	RMSE	Average of Accuracy (%)	RMSE	Accuracy (%)	RMSE
Dataset I	98.723	0.001	96.331	0.005	94.604	0.015	96.553	0.007
Dataset II	64.605	0.120	47.482	0.006	76.259	0.005	62.782	0.044
Dataset III	95.370	0.023	84.299	0.047	82.104	0.008	87.258	0.026
Dataset IV	93.957	0.063	93.237	0.029	87.691	0.020	91.628	0.037

### 3.3. Experiment II

**Figure 7:**
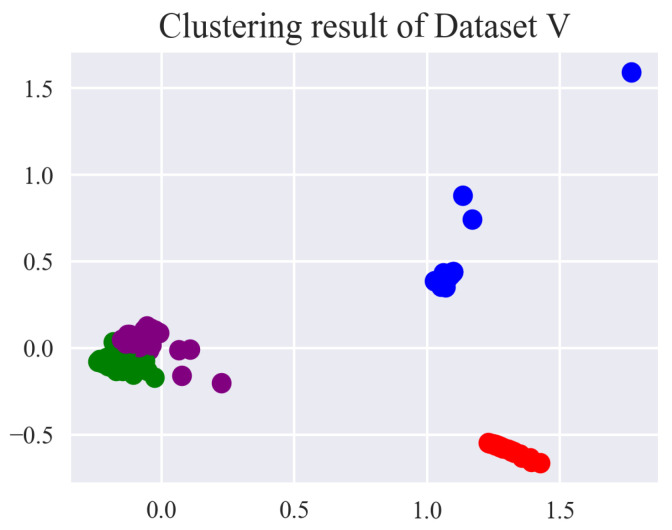
Clustering result for Dataset V after mini batch k-means.

To verify whether the features obtained after eliminating redundant features with mRMR would have better performance on the clustering effect, the remaining features except for the selected features (Dataset I) with mRMR composed Dataset V. Table 10 lists the features of Dataset V. Similarly, the DBIs with the clustering number ranging from 2 to 10 by mini batch k-means are calculated in Table 11. As shown in Figure 7, Dataset V was unable to determine the numbers of the right clusters.

**Figure 8:**
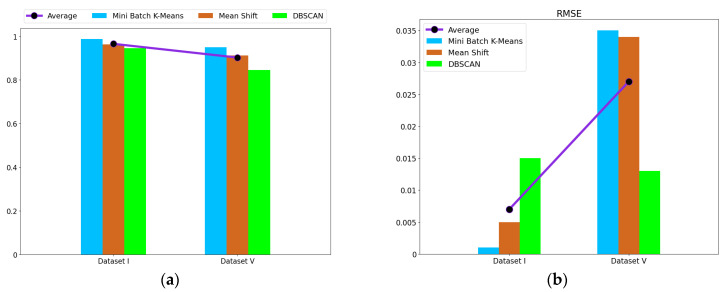
Display of the diagnosis results for different datasets after clustering: (**a**) average of accuracy; (**b**) RMSE.

The experiment results were obtained by the average of 10 trials based on mini batch k-means. The validity of the proposed method was compared with different clustering methods. The diagnosis results for different datasets after clustering are shown in Table 12. Mini batch K-means had the best accuracy and the lowest RMSE compared with the other two clustering methods. As shown in Figure 8, the redundant feature information removed affects the clustering, and the features of Dataset I selected by mRMR are the optimal fault features.

**Table 12:** Average of accuracy and RMSE for different datasets after clustering.

Different Datasets	Mini Batch K-Means		Mean Shift		DBSCAN		Average	
	Average of Accuracy (%)	RMSE	Average of Accuracy (%)	RMSE	Average of Accuracy (%)	RMSE	Accuracy (%)	RMSE
Dataset I	98.723	0.001	96.331	0.005	94.604	0.015	96.553	0.007
Dataset V	95.018	0.035	91.259	0.034	84.514	0.013	90.264	0.027

### 3.4. Experiment III

**Figure 9:**
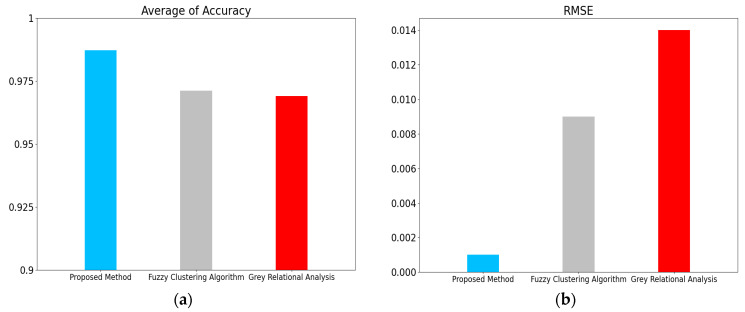
Display of the diagnosis results for different methods: (**a**) average of accuracy; (**b**) RMSE.

**Figure 10:**
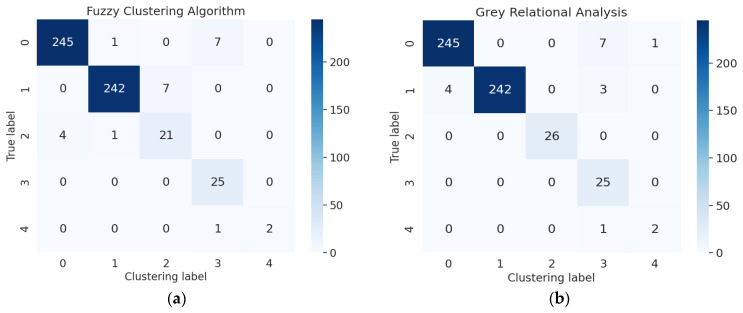
Confusion matrixes for different methods: (**a**) fuzzy clustering algorithm; (**b**) grey relational analysis.

There is little research on fault detection methods for electrohydraulic switch machines now. This paper compared the proposed diagnosis method with the existing techniques for a fuzzy clustering algorithm [20] and grey relational analysis [24]. We changed the parameters of the Hamming distance method for a fuzzy clustering algorithm and the resolution ratio of the relational coefficient for grey relational analysis 10 times. The average of accuracy and RMSE are calculated in Table 13. The diagnosis results for different methods are shown in Figure 9. The confusion matrixes for different methods are shown in Figure 10. Experiment results showed that this proposed method provided higher accuracy and proved the effectiveness of the fault identification task for the electrohydraulic switch machine.

**Table 13:** Average of accuracy and RMSE for different methods.

Different Methods	Average of Accuracy (%)	RMSE
Proposed method	98.723	0.001
Fuzzy clustering algorithm	97.122	0.009
Grey relational analysis	96.908	0.014

### 3.5. Experiment Results Analysis

The experimental comparisons showed that the sectionalized feature extraction method according to the three action stages (30%–60%–10%) of a switch machine is more robust and effective for fault clustering than other methods. On the basis of the above sectionalization mode, it is necessary to choose the optimal features, and the most effective features can be singled out by mRMR method. Finally, the proposed diagnosis method of electrohydraulic switch machines based on mini batch k-means had the lowest misdiagnosis rate compared with other existing methods.

Therefore, it can be found that the proposed intelligent fault detection method can not only get the optimal features and identify different action states, but also detect faults at a higher accuracy rate. The effectiveness of the proposed sectionalized feature extraction method is verified successfully by experiments.

## 4. Conclusions

The fault detection of the electrohydraulic switch machine based on sectionalized feature extraction from the oil pressure signal during the working process was proposed in this paper. The fault detection framework for the electrohydraulic switch machine was established. First, the whole oil pressure signal was divided into three stages with the sectionalization time ratio (30%–60%–10%) according to the switching process. Five features of each stage of the oil pressure signal were computed, and the features were smoothed and normalized. Then, the mRMR algorithm was used to eliminate redundant features and derive the optimal feature set. The mini batch k-means clustering method was applied to achieve the fault detection of the electrohydraulic switch machine. The contrastive experiments verified the effectiveness and accuracy of the proposed method.

We came to the conclusion that (1) the mean, peak-to-average ratio (PAR), and impulse (IM) of the oil pressure signal interval stage were superior to other types of features for the fault identification of an electrohydraulic switch machine, whereas kurtosis (Ku) and variance (Var) did not perform as well in terms of fault clustering. (2) Moreover, the features extracted from the unlocking and locking stages outnumbered those of the conversion stage, which meant the different fault information in each stage. (3) The fault detection method proposed in this paper, which was based on the unsupervised algorithm, can realize free-label fault detection in high recognition. The proposed method can provide support for railway staff without the cost of massive sample data. The experiments verified that the proposed diagnosis method could accurately identify faults of electrohydraulic switch machines and reduce economic costs. Due to the limited fault sample data collected in this paper, we will acquire more comprehensive fault sample data as much as possible in the next step to further increase the accuracy of the method. In the future, we will improve the method to fully meet the needs of practical engineering application.

## Figures and Tables

**Table 2:** Mechanical fault types and cause analysis of an electrohydraulic switch machine.

Fault Types		Fault Cause Analysis
Mechanical Faults	Not locking or not unlocking	The switch rail is stuck with the basic rail; the sliding board is severely suspended and the friction force is large; the lock hook and the action rod are severely rubbed.
	There is abnormal resistance in the unlocking phase	There are a few foreign objects and insufficient oil injection on the normal position locking position of locking iron.
	There is abnormal resistance in the locking phase	There are a few foreign objects and insufficient oil injection on the reverse position locking position of locking iron.
	There is a mechanical jam during the conversion phase	The stiffness degradation of the outer locking device is too severe, or there is a foreign matter on the sliding board, which causes excessive resistance of the turnout during conversion.
	Unclosed contact point causes loss of turnout representation	The gap adjustment does not meet the standard, which causes the fault of the moving contact conversion.

**Table 4:** The feature set of Dataset I after mRMR.

Dataset I	Interval Stage	Feature Types
Left oil pressure	Segment I	Var/IM
	Segment II	
	Segment III	Mean/Ku/PAR
Right oil pressure	Segment I	Mean/IM
	Segment II	Mean/PAR
	Segment III	PAR

**Table 5:** The feature set of Dataset II after mRMR.

Dataset II	Interval Stage	Feature Types
Left oil pressure	Whole	Mean/PAR/IM
Right oil pressure	Whole	IM

**Table 6:** The feature set of Dataset III after mRMR.

Dataset III	Interval Stage	Feature Types
Left oil pressure	Segment I	Mean
	Segment II	Mean/Var
Right oil pressure	Segment I	Var/PAR
	Segment II	Ku/IM

**Table 7:** The feature set of Dataset IV after mRMR.

Dataset IV	Interval Stage	Feature Types
Left oil pressure	Segment I	Mean/PAR
	Segment II	Ku/PAR/IM
	Segment III	
Right oil pressure	Segment I	PAR/IM
	Segment II	Mean/PAR/IM
	Segment III	

**Table 8:** Variations of the DBIs with the number of clusters for different datasets.

DBI	The Number of Clusters								
	2	3	4	5	6	7	8	9	10
Dataset I	0.400	0.206	0.221	0.201	0.290	0.348	0.408	0.459	0.855
Dataset II	0.795	0.630	0.465	0.433	0.302	0.344	0.380	0.524	0.551
Dataset III	0.248	0.181	0.158	0.286	0.372	0.583	0.369	0.351	0.437
Dataset IV	0.777	0.173	0.102	0.281	0.500	0.947	0.691	0.812	0.573

**Table 10:** The feature set of Dataset V after mRMR.

Dataset V	Interval Stage	Feature Types
Left oil pressure	Segment I	Mean/Ku/PAR
	Segment II	Mean/Var/Ku/PAR/IM
	Segment III	Var/IM
Right oil pressure	Segment I	Var/Ku/PAR
	Segment II	Var/Ku/IM
	Segment III	Mean/Var/Ku/IM

**Table 11:** Variations of the DBIs with the number of clusters for different datasets.

DBI	Number of Clusters								
	2	3	4	5	6	7	8	9	10
Dataset I	0.400	0.206	0.221	0.201	0.290	0.348	0.408	0.459	0.855
Dataset V	0.504	0.187	0.182	0.286	0.742	0.799	0.665	0.718	0.940

## Data Availability

The data used in this study are all owned by the research group and will not be transmitted.

## Abbreviations

The following abbreviations are used in this manuscript:

ANN	artificial neural network
CHI	Calinski–Harabaz index
DBI	Davies–Bouldin index
DWST	deep wavelet scattering transform
EEMD	ensemble empirical mode decomposition
HSMMs	hidden semi-Markov models
IM	impulse
KFCM	kernel fuzzy c-means
Ku	kurtosis
LLE	local linear embedding
LPP	local preserving projection
LSTM	long short-term memory
LWR	locally weighted regression
MI	mutual information
mRMR	max-relevance and min-redundancy
PAR	peak-to-average ratio
PCA	principal component analysis
SVM	support vector machine
Var	variance
VMD	variational mode decomposition
